# Spatial multiomics in biomedical research: advances beyond transcriptomics

**DOI:** 10.1172/jci.insight.206951

**Published:** 2026-09-08

**Authors:** Xinchen Mao, Zhuo Chen, Emily J. Hwang, Jun Liu, Junrou Huang, Haikuo Li

**Affiliations:** 1 Precision Research Center for Refractory Diseases, Shanghai Jiao Tong University Pioneer Research Institute for Molecular and Cell Therapies, Shanghai General Hospital, Shanghai Jiao Tong University School of Medicine, Shanghai, China.; 2 Undergraduate Program in Molecular, Cellular, and Developmental Biology, Yale University, New Haven, Connecticut, USA.; 3 Department of Nephrology, Shanghai General Hospital, Shanghai Jiao Tong University School of Medicine, Shanghai, China.; 4 College of Biological Science and Medical Engineering, Donghua University, Shanghai, China.; 5 State Key Laboratory of Innovative Immunotherapy, School of Pharmaceutical Sciences, Shanghai Jiao Tong University, Shanghai, China.

## Abstract

Coordinated changes in gene expression, epigenetic regulation, protein and metabolic activities together drive disease progression and determine clinical outcomes. While spatially resolved transcriptomics has been widely adopted across biomedical fields, it offers an incomplete picture limited to transcriptomic levels. Here, we survey the latest developments in spatial multiomics technologies, with particular emphasis on platforms that extend beyond conventional transcriptomics and profile genomics, epigenomics, proteomics, or metabolomics within intact tissues. These approaches are rapidly becoming commercialized, and here we highlight major technical breakthroughs, enhanced sample compatibility, emerging applications, and computational tools for data analysis. This Review aims to equip researchers with a clear understanding of the current technological landscape and to accelerate the adoption of spatial multiomics methods in biomedical research.

## Introduction

In human pathophysiology, cell-state transitions, defined by coordinated changes in gene expression, epigenetic modifications, and protein and metabolic profiles, drive disease progression and determine clinical outcomes. The intrinsic cellular heterogeneity within tissues has hindered efforts to unravel these complex, spatially organized processes. Recently, single-cell multiomic technologies have progressed from transcriptome-only profiling to multimodal measurements within individual cells, thereby providing a bridge from central dogma to revealing complex regulatory relationships between genome, epigenome, transcriptome, and proteome. These advances have profoundly illuminated cell heterogeneity and its pathophysiological relevance in both rodent models and human tissues (1). Conventional single-cell approaches, while informative, require tissue dissociation, resulting in complete loss of spatial context. This limitation is critical, as precise positional information governs cell-cell interactions, disease niche formation, and emergent cellular phenotypes in diverse human diseases.

Spatial transcriptomics (STs) has now matured into a fully commercialized and widely adopted platform that preserves tissue architecture while enabling genome-scale gene expression mapping (2). For unbiased sequencing, the 10× Genomics Visium and Visium HD were widely adopted due to their robust compatibility with standard clinical histology workflows, including formalin-fixed, paraffin-embedded (FFPE) tissues. Stereo-seq delivers subcellular resolution across exceptionally large fields of view. Imaging-based platforms like the 10× Genomics Xenium map thousands of targeted transcripts at single-molecule resolution. As of today, ST has powered the construction of comprehensive transcriptomic atlases and uncovered previously unrecognized cellular neighborhoods in different tissues. Despite these successes, transcriptomics alone offers an incomplete picture, as mRNA levels frequently correlate poorly with protein abundance or epigenetic state (3) and do not directly reflect processes central to physiology, such as metabolic activities and small molecule handling (4).

**Figure 1:**
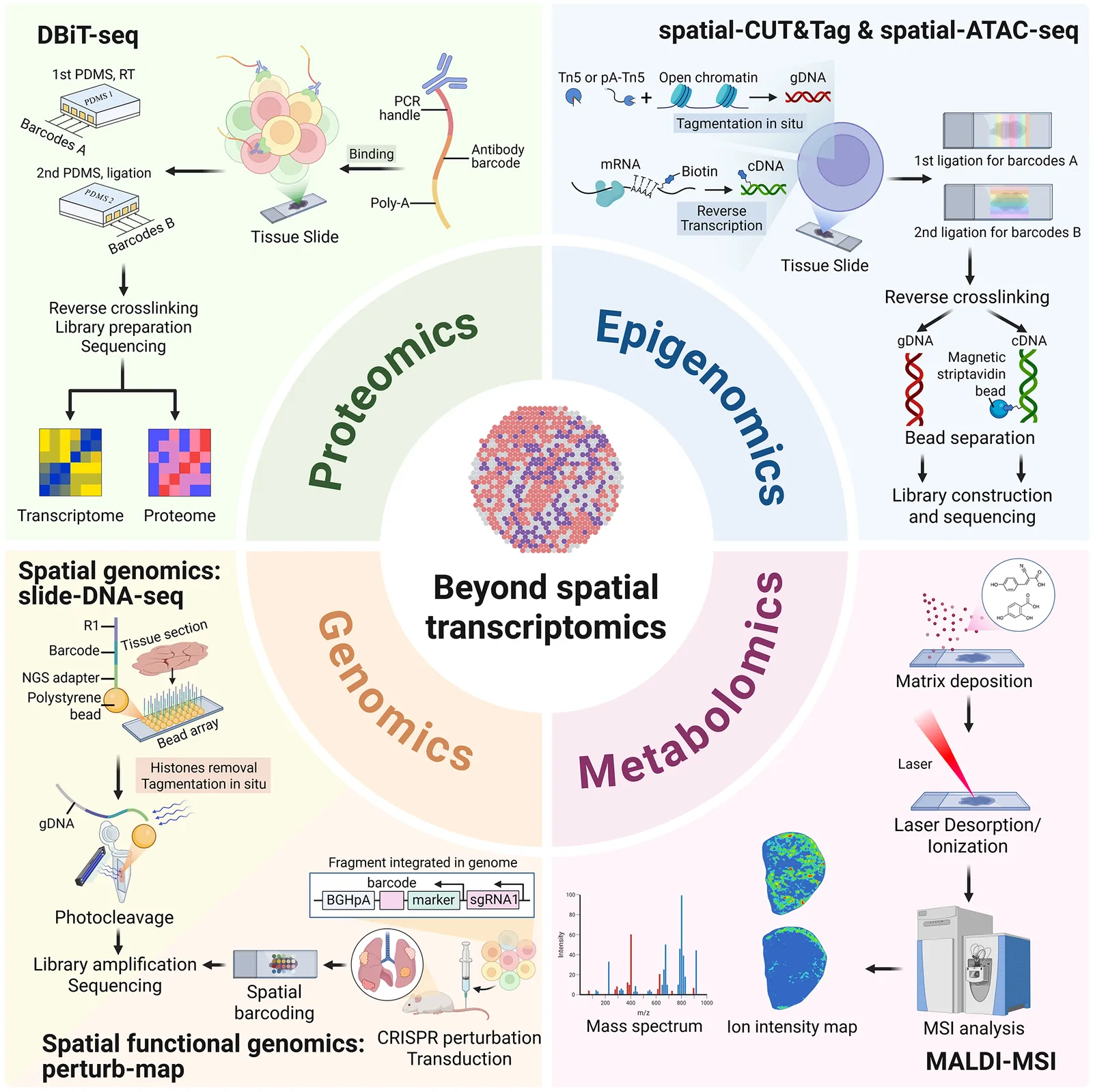
Representative spatial omics technologies for non-transcriptome modalities and their simplified workflows. Deterministic barcoding in tissue sequencing (DBiT-seq) (9): Co-profiling of protein Ab-derived tags and transcripts on a microfluidic platform. Spatially resolved profiling of histone modifications using in situ cleavage under targets and tagmentation (spatial-CUT&Tag) (14) and spatially resolved assay for transposase-accessible chromatin using sequencing (spatial-ATAC-seq) (13): Analysis of chromatin-associated protein binding and chromatin accessibility profiles with the DBiT-seq microfluidic platform. The illustration presents spatial-CUT&Tag-RNA-seq and spatial-ATAC-RNA-seq (58, 60), which enable simultaneous profiling of the epigenome and transcriptome. Slide-DNA-seq (40): Spatially resolved genome sequencing using microbead arrays with unique DNA barcodes. Perturb-map (46): Spatial functional genomics based on CRISPR/Cas9 gene modification. MALDI mass spectrometry imaging (MALDI-MSI) (21): MS-based spatial metabolomics technology achieved through spot laser irradiation. PDMS, polydimethylsiloxane; RT, reverse transcription; NGS, next-generation sequencing.

Recognizing these gaps, the field is experiencing rapid growth in spatial multiomics technologies that sequentially or simultaneously capture distinct molecular layers (genome sequence, chromatin accessibility, histone modifications, transcriptome, proteome, and metabolome) within intact tissues (Figure 1). These emerging approaches promise to deliver far richer, functionally informative maps of organ organization and dysfunction in both steady and diseased states than transcriptomics alone. In this Review, we systematically survey cutting-edge spatially resolved multiomics technologies and highlight recent major breakthroughs, technical principles, current performance, and landmark applications (Tables 1, 2, 3, and Supplemental Table 1; supplemental material available online with this article; https://doi.org/10.1172/jci.insight.206951DS1). Additionally, we discuss computational tools and integrative frameworks designed for spatial multiomics data analysis.

## Spatial omics methodology

**Figure 2:**
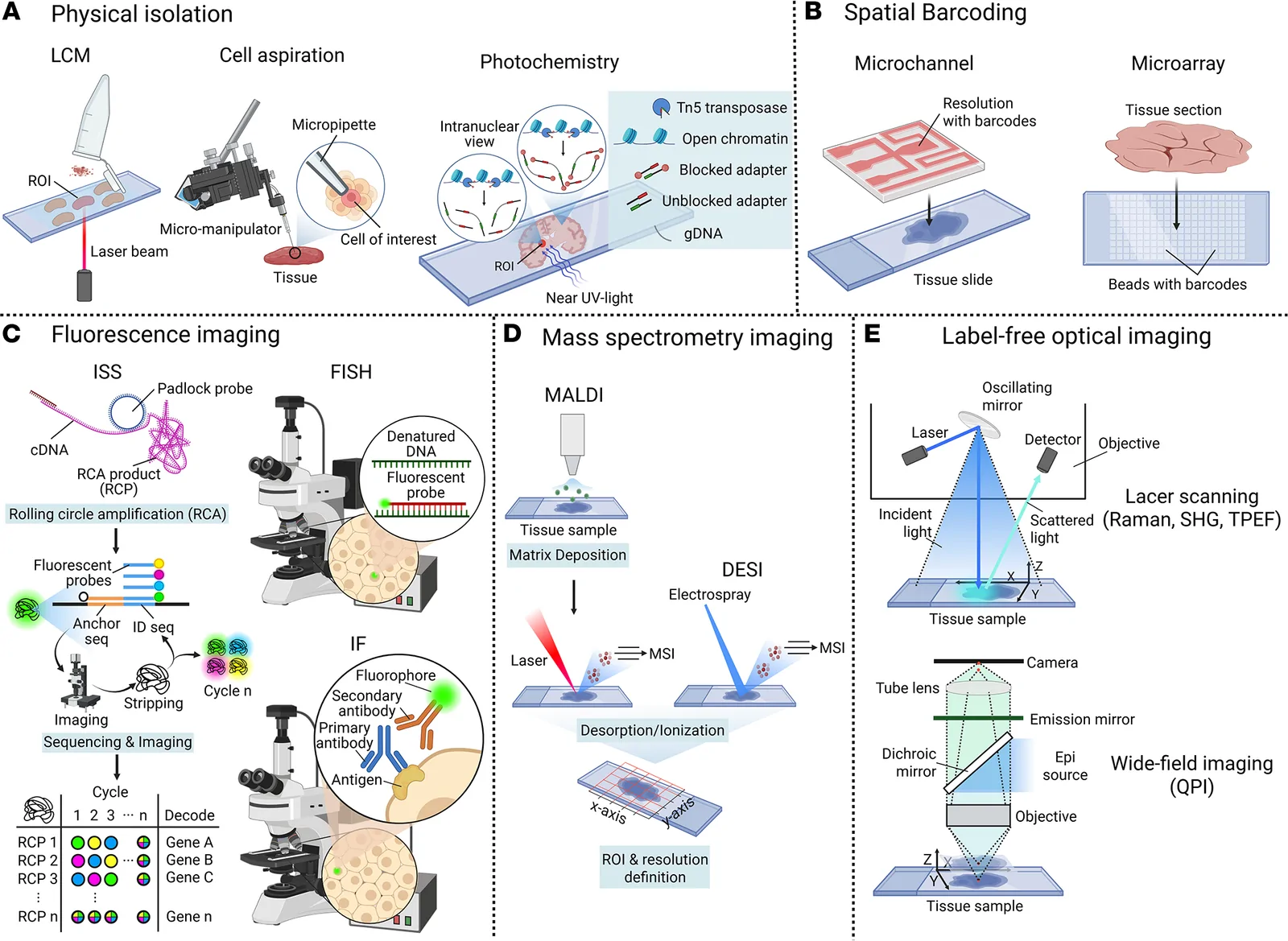
Principles of spatial omics technologies. (**A**) Physical isolation can be broadly categorized into three main approaches: laser capture microdissection (LCM) of regions of interest (ROIs) (55), direct aspiration of target cells using a micropipette guided by a micromanipulator (6), and unphotocaging of ROIs through light of a specific wavelength (5). Specifically, the photochemical approach selects ROIs by photocaging and uncaging of photocleavable adapters on the nucleic acid molecule. (**B**) Spatial barcoding typically constructs a 2D mosaic composed of multiple pixels via microfluidic channels (9) or microbead arrays (82) that each carry a unique DNA barcode. Subsequently, cells and their contents within each pixel are tagged with a unique spatial coordinate. (**C**) Fluorescence imaging directly visualizes the spatial location of target molecules through in situ fluorescence imaging. (**D**) MS imaging ionizes molecules in the sample using a laser beam (MALDI) or electrospray (desorption electrospray ionization, DESI). (**E**) Label-free optical imaging. It captures specific molecules by detecting transmitted light, scattered light, exciting light, or intrinsic emission from cells or tissues, without the need for staining. QPI, quantitative phase imaging; SHG, second harmonic generation; TPEF, two-photon epifluorescence.

Like single-cell omics, spatial multiomics technologies also rely on distinct platforms that enable spatially resolved profiling within intact tissue sections. Representative spatial multiomics techniques are summarized in Tables 1–3 and Supplemental Tables 1 and 2 along with technical specifications and current biomedical research applications. Depending on their underlying principles, current spatial multiomics techniques can be classified into the following five major categories: (a) Physical isolation followed by sequencing of regions of interest (ROIs), e.g., photoselective sequencing (PSS) (5) and Image-seq (6) (Figure 2A). (b) Spatial barcoding using in situ hybridization of spatially indexed nucleotide sequences (barcodes) with intracellular molecules (Figure 2B). These identical barcodes vary across coordinates, allowing computational reconstruction of a 2D tissue molecular mosaic. Such spatial barcoding can be implemented via either microbeads or microfluidic arrays, e.g., Visium HD (7), Stereo-seq (8), deterministic barcoding in tissue for spatial omics sequencing (DBiT-seq) (9), Stereo-seq v2 (10), Array-seq (11), spatial co-indexing of transcriptomes and epitopes (spatial-CITE-seq) (12), spatially resolved assay for transposase-accessible chromatin using sequencing (spatial-ATAC-seq) (13), spatially resolved profiling of histone modifications using in situ cleavage under targets and tagmentation (spatial-CUT&Tag) (14), and spatial DNA methylome-transcriptome sequencing (spatial-DMT) (15) (Figure 2C). (c) Fluorescence imaging leverages Abs or nucleotide sequences (e.g., in situ sequencing [ISS]) conjugated to fluorophores to target molecules of interest in tissue. There are three major methodology categories of fluorescence sequencing: ISS, in which fluorescence signals are first amplified via padlock probe hybridization and rolling circle amplification, followed by multiple rounds of sequence-specific cyclic fluorescence imaging. This approach facilitates direct readout of nucleic acid sequences within tissue sections (e.g., ExSeq, STARmap PLUS, and oligoFISSEQ) (16–18); FISH (e.g., CosMx [ref. 19] and EEL FISH); and immunofluorescence (IF) (e.g., iterative bleaching extension [IBEX]; ref. 20) (Figure 2D). (d) Mass spectrometry imaging (MSI) achieves spatial resolution by scanning tissue sections pixel-by-pixel, ionizing molecules for MS analysis. The predominant ionization techniques include matrix-assisted laser desorption/ionization (MALDI) and desorption electrospray ionization (DESI) (e.g., MALDI-MSI and DESI-MSI; refs. 21, 22) (Figure 2E). (e) Label-free optical imaging acquires raw images from varying illumination angles and focal planes (e.g., multimodal Raman-based platforms; ref. 23) and employs computational algorithms for imaging integration and processing.

## Recent advancements in ST

ST has transformed tissue biology by enabling gene expression to be mapped within intact anatomical contexts, thereby redefining how cellular identity, niche organization, and microenvironmental signaling are interpreted in situ. ST technologies can also be broadly divided into two major categories: imaging-based and sequencing-based approaches. This section focuses on emerging ST technologies introduced over the past two to three years, many of which achieve substantially higher resolution, sample compatibility, or multimodal capabilities, than previous approaches. A comparison between conventional transcriptomic approaches and representative commercial ST platforms is provided in Supplemental Table 2.

Imaging-based ST approaches directly visualize RNA molecules through FISH or ISS. Commercially available platforms such as the 10× Genomics Xenium (24) belong to this category, combining highly multiplexed RNA FISH with automated imaging and computational cell segmentation to achieve subcellular-resolution transcript mapping within intact tissues. New variants, such as EEL FISH (25), which electrophoretically transfers RNA onto a glass surface to reduce tissue autofluorescence, further enhance detection sensitivity and spatial fidelity. ISS methods extend these capabilities by sequencing barcoded probes hybridized to RNA or cDNA in situ. Examples include ExSeq (16) for cDNA-based detection, and STARmap PLUS (17) for direct RNA sequencing (RNA-seq). Recently, Wan et al. developed whole-embryo MERFISH (weMERFISH) (26), which achieved subcellular-resolution ST mapping across entire zebrafish embryos and established a whole-organism atlas that integrates spatial gene expression, chromatin accessibility, and morphogenetic dynamics. Recent studies further demonstrate the scalability and clinical applicability of MERFISH, including large-scale FFPE profiling of more than 60 human heart specimens (27) and the development of MERFISH+ (28), which expands transcript detection throughput and multiplexing capacity.

Sequencing-based ST approaches rely on spatially barcoded arrays or microfluidic channels to assign spatial coordinates to captured mRNAs. Representative platforms include the 10× Genomics Visium HD (7), Stereo-seq v2 (10), Pixel-seq (29), and DBiT-seq (9). Visium HD represents a widely adopted commercial platform that combines transcriptome-wide RNA capture with high-density spatial barcoding, providing near-cellular spatial resolution while maintaining broad compatibility with standard histological workflows. Among these, DBiT-seq stands out for its high spatial resolution and tissue compatibility. The original DBiT-seq achieves near-single-cell resolution using microfluidic barcoding workflows that work with fresh-frozen tissues. A recent pivotal advance for clinical translation is Patho-DBiT (30) (Table 2), which was specifically developed to enable whole-transcriptome (total RNA) profiling from archival FFPE samples with degraded RNA. By integrating in situ polyadenylation and optimized crosslink-reversal chemistry, Patho-DBiT overcomes longstanding RNA degradation barriers in FFPE samples and accelerates the application of ST to routine clinical specimens, thereby enabling comprehensive analysis of diverse RNA species, including mRNA, miRNA, tRNA, and splicing isoforms. Recently, the development of DBiTplus (31) further extends the platform by co-mapping whole-transcriptome data with highly multiplexed CODEX-based proteomics on the same tissue section. However, despite these advances, spatial profiling of miRNAs remains technically challenging. The short length of mature miRNAs restricts probe design and increases the risk of off-target hybridization, while distinguishing primary, precursor, and mature miRNA species within intact tissues remains difficult. Consequently, further methodological improvements will be required to achieve sensitive and specific spatial small-RNA profiling in clinical samples (32).

Additionally, Cipurko et al. introduced Array-seq (11), which repurposes conventional oligonucleotide microarrays by embedding unique barcodes at known coordinates and performs a simple two-step mRNA capture reaction. Alternatively, Seq-Scope (33) leverages the Illumina sequencing infrastructure for ST by converting the flow cell into a chip with unique spatial barcodes generated through next-generation sequencing. More recently, Open-ST (34) was introduced as an open-source sequencing-based framework that combines subcellular-resolution transcriptomics with scalable 3D tissue reconstruction, expanding the accessibility of high-resolution ST.

## Spatial genomics

The term “genomics” generally encompasses multiple omics layers. For clarity, we will restrict “spatial genomics” to technologies that directly interrogate genomic DNA. Currently, these approaches broadly fall into two major categories: imaging-based methods and sequencing-based methods.

Imaging-based spatial genomics comprises FISH-based methods (e.g., DNA-MERFISH and DNA seqFISH+; refs. 35, 36) and ISS-based approaches (e.g., OligoFISSEQ; ref. 18) (Table 1). Common approaches like Hi-C (37) lack direct in situ visualization of chromatin organization and cannot achieve single-cell resolution. Meanwhile, conventional FISH and live-cell CRISPR imaging are channel limited, typically permitting the observation of at most a dozen loci simultaneously. Consequently, a panoramic view of entire chromosomes with genome-wide coverage has remained elusive. The biological purpose of the technologies mentioned above is to characterize the spatial organization of genomic loci and subnuclear structures within single cells. The resolution of genome-wide targeting resolution has been progressively refined from 1 Mb to 25 kb, whereas the single-gene resolution spans from tens to hundreds of kilobases (18, 35, 36, 38). Several excellent reviews have previously described the technical details of these methods (39). Notably, the advanced version of DNA seqFISH+, known as 2-layer DNA seqFISH+ (38), enhances resolution to the sub-megabase scale. In this method, the genome is first partitioned into multiple chromosomal blocks. The initial rounds of imaging resolve the relative positions of sub-units within these blocks and specific genomic coordinates of the blocks can be resolved in subsequent analysis. This approach achieves genome-wide coverage of over 100,000 DNA loci through a two-layer barcoding method.

Combined with RNA seqFISH+ and sequential IF, nascent transcripts for 17,856 genes and subnuclear markers have been mapped in adult mouse cerebellum cells (38). Due to the reliance on high-magnification microscopy, imaging-based techniques are inherently constrained by high resolution, which render them less suitable for tissue-wide analysis.

Sequencing-based spatial genomics relies on spatial barcoding to achieve spatially resolved DNA sequencing at the intact-tissue scale. Unlike imaging-based technologies that specifically target candidate genes, the sequencing-based approaches unbiasedly capture the whole genome unbiasedly through mechanisms such as poly-T probes immobilized on the surfaces of beads or chips, which facilitates the discovery of cellular heterogeneity. Slide-DNA-seq (40) (Figure 1) employs a monolayer of barcoded beads on a glass slide to spatially resolve genomic DNA fragments. Multiple displacement amplification (MDA) is widely applied in whole genome amplification; however, its use in large-scale spatial genomics is restricted due to high cost, limited throughput, and amplification bias. To overcome these limitations, barcoded MDA (bMDA) (41) integrates cell barcodes, enabling cost-effective, whole-genome spatial detection at single-nucleotide resolution, even in a very limited microenvironment. In the context of precision medicine, sequencing-based spatial genomics enables the localization of distinct tumor clones within tissues and the de novo discovery of cell-intrinsic genetic aberrations simultaneously. Zhao et al. applied slide-DNA-seq to the a mouse model of liver metastasis and profiled copy number alterations (CNAs), and stratified cancer cells into three subclusters characterized by distinct spatial and transcriptomic features. By integrating with ST, they quantitatively decoupled the regulatory mechanisms governing the tumor transcriptome, unearthing three distinct categories of gene programs: genetically driven, environment-sensitive, and dually driven (40). Kim et al. leveraged bMDA to simultaneously detect CNAs, single-nucleotide variants, structural variations, and kataegis, based on which the study reconstructed the evolutionary relationships of tumor clone populations within a spatial context (41). Both approaches have effectively characterized the clonal heterogeneity and the spatial epigenetic landscape of human tumors, including colon cancer and triple-negative breast cancer (40, 41).

Recently, hybrid techniques that coordinate both imaging-based and sequencing-based technologies have emerged. In situ genome sequencing (IGS) (42) combines in situ imaging with ex situ sequencing to simultaneously obtain unbiased localization and sequence information of specific genome fragments at a subnuclear scale. However, standalone IGS is restricted by the optical diffraction limit and the confined space of the cell nucleus. Integration of the expansion microscopy technique yielded ExIGS (43), which further enhances both genomic coverage and spatial resolution (to nanometers).

Of note, spatial functional genomics methods have been developed in the past few years. Functional genomics investigates phenotypic variations after genotypic perturbations (44) (Figure 1). For example, Perturb-seq (45) is a pioneering single-cell functional genomics method that combines CRISPR technology with single-cell RNA-seq (scRNA-seq), which is sufficiently accessible to drive functional genomics toward high-throughput, decentralization, and dissection of intricate gene regulatory networks. This concept has successfully translated into the spatial domain through innovative platforms, including Perturb-map (46), Perturb-Multi (47), and PERTURB-CAST (48). Perturb-Multi has allowed investigation of key pathways regulating zonation, ER stress, and steatosis in mouse hepatocytes (47), while PERTURB-CAST has screened 256 candidate cancer-driving genotypes and evaluated eight combinatorial perturbations in mouse liver cancer (48). Notably, Perturb-map has analyzed the impacts of a series of genes’ deletion on tumor morphology, stromal differentiation, and the tumor microenvironment (TME). *Tgfbr2* gene knockdown triggers T cell spatial exclusion, which offers a putative biomarker for identifying immunologically cold tumors (46). In subsequent studies, IL-4 secreted by ovarian cancer cells drives neighboring macrophages toward the protumor M2-like phenotype, thereby conferring resistance to anti–PD-1 immunotherapy (49). As such, genetic ablation of IL-4 in tumor cells directly reverses this effect. These proof-of-concept studies highlight the transformative potential of spatial functional genomics (50).

## Spatial epigenomics

Gene transcription is tightly controlled by multiple layers of epigenetic information, including DNA methylation, posttranslational histone modifications, chromatin accessibility, and higher-order 3D genome organization (51). The epigenome is highly susceptible to homeostatic and developmental fluctuations, thus directly reflecting long-term stability and plasticity of cell fate.

Over the past decade, single-cell epigenomics has matured to robustly profile chromatin accessibility, histone modifications, DNA methylation, and 3D genome conformation (52). However, a cell’s epigenetic state is inextricably coupled with its surroundings, and it serves as a critical nexus for deciphering how environmental perturbations shape the transcriptome. For instance, malignancies and fibrotic remodeling following organ injury exhibit profound heterogeneity and microenvironmental dependency. Consequently, the need for epigenome profiling epigenome within native tissues has catalyzed the emergence of spatial epigenomics.

Transposon 5 (Tn5) transposase has revolutionized modern epigenomics through its highly efficient tagmentation mechanism, whereby hyperactive Tn5 mutants simultaneously fragment DNA and insert sequencing adapters (Supplemental Figure 1). This process dramatically reduces input requirements, hands-on time, and amplification bias compared with traditional enzymatic assays (53), while offering inherent compatibility with multimodal readouts, making it the cornerstone of nearly all spatial epigenomics technologies.

Two representative spatial epigenomic methods are spatial-ATAC-seq (13) and spatial-CUT&Tag (14). Applied to mouse embryos and brains, both techniques successfully mapped their epigenetic landscapes at the tissue level (13, 14). Specifically, spatial-ATAC-seq identified 2 spatially nested, epigenetically distinct TMEs in human glioblastoma tissues, revealing a novel mechanism whereby interlocking spatial gradients of short- and long-range signals released by each niche drive tumor cell plasticity through the dynamic remodeling of chromatin accessibility (54). Both methods combine Tn5-mediated tagmentation with microfluidic deterministic barcoding to map open chromatin or specific histone modifications at near-single-cell resolution across tissue sections (Supplemental Figure 1). Complementary approaches include laser-capture microdissection coupled to ATAC-seq (LCM-ATAC-seq) (55) and light-activated library construction for ROIs (PSS) (5) (Figure 2).

Llorens-Bobadilla et al. (56) proposed that spatial context serves as the primary driver of chromatin state heterogeneity. They utilized solid-phase capture with barcoded microarrays, and this spatial ATAC method enhanced throughput and coverage over previous microfluidic techniques. Approximately 6,000 distal regulatory elements were mapped, and their activation patterns strictly aligned with tissue-specific domains, such as liver hematopoietic and cardiac myocardial regions. Additionally, they reported continuous spatial epigenomic remodeling in the transition from SOX2^+^ progenitor cells to mature neurons.

Wang et al. (57) applied spatial-ATAC-seq profiling of non-functional pancreatic neuroendocrine tumors (NF-PanNETs) and revealed 2 divergent tumor-stroma niches, a proliferative niche driven by MYC/FOX transcription factors (TFs) and an invasive niche defined by SNAIL TFs and KRAS-mediated epithelial-mesenchymal transition programs. Moreover, myofibroblastic cancer-associated fibroblasts in the TME differentially shape tumor behavior through neighboring pro-proliferative interactions and distal pro-invasive signaling, highlighting the role of spatial context in epigenomic heterogeneity (57).

The flexibility of Tn5 tagmentation has enabled the transition from single modalities to true spatial multiomics. Several platforms now jointly profile the epigenome and transcriptome from the same tissue section using a shared workflow, where in situ tagmentation and reverse transcription are first performed, after which genomic DNA and cDNA receive identical spatial barcodes before modality-specific library separation (15, 58–60). Building on the DBiT-seq platform (Figure 2), this design underlies spatial-ATAC–RNA-seq, spatial-CUT&Tag-RNA-seq (58, 60), spatial-DMT (15), and MISAR-seq (59). Two tri-omics technologies, spatial-ATAC-RNA-protein-seq (spatial-ARP-seq) and spatial-CUT&Tag-RNA-protein-seq (spatial-CTRP-seq) (61), have recently been developed to further incorporate Ab-derived tags to simultaneously capture chromatin accessibility or histone modifications, the whole transcriptome, and up to 150 proteins. Similarly, spatial-Mux-seq (62) achieves concurrent mapping of transcriptome, chromatin accessibility, and multiple histone modifications. These highly integrated, scalable workflows, which have been validated in mouse embryos and brain tissues, represent a clear path toward comprehensive, unbiased spatial atlases that span the central dogma within an intact tissue. These technologies are anticipated to witness more large-scale and in-depth applications in the future.

Of note, a major limitation of most spatial epigenomics methods has been the requirement of fresh-frozen samples. Recent studies attempted to overcome this barrier by optimizing antigen-retrieval and decrosslinking conditions, enabling improved chromatin accessibility profiling from archival FFPE specimens such as human cerebellum, thymus, lymphoma, and melanoma (63, 64). This advance unlocks vast clinical biobanks and routine diagnostic samples, creating unprecedented opportunities for large-scale, retrospective spatial epigenomics studies. Although less common, imaging-based epigenomics approaches like DNA-encoded amplifying FISH (DEA-FISH) (65) and epigenomic MERFISH (66) are also emerging. They are mainly utilized to observe subnuclear and chromosomal organization and for the detection of low-abundance histone modifications and nucleic acid marks through in situ amplification and high-resolution imaging.

The incorporation of epigenomics into spatial multiomics holds transformative potential. As spatial epigenomics platforms are becoming commercially available (54), these tools are no longer restricted to specialized laboratories and will enable integration of native epigenomic profiling into the next generation of atlas construction and clinical-translational studies.

## Spatial proteomics

**Figure 3:**
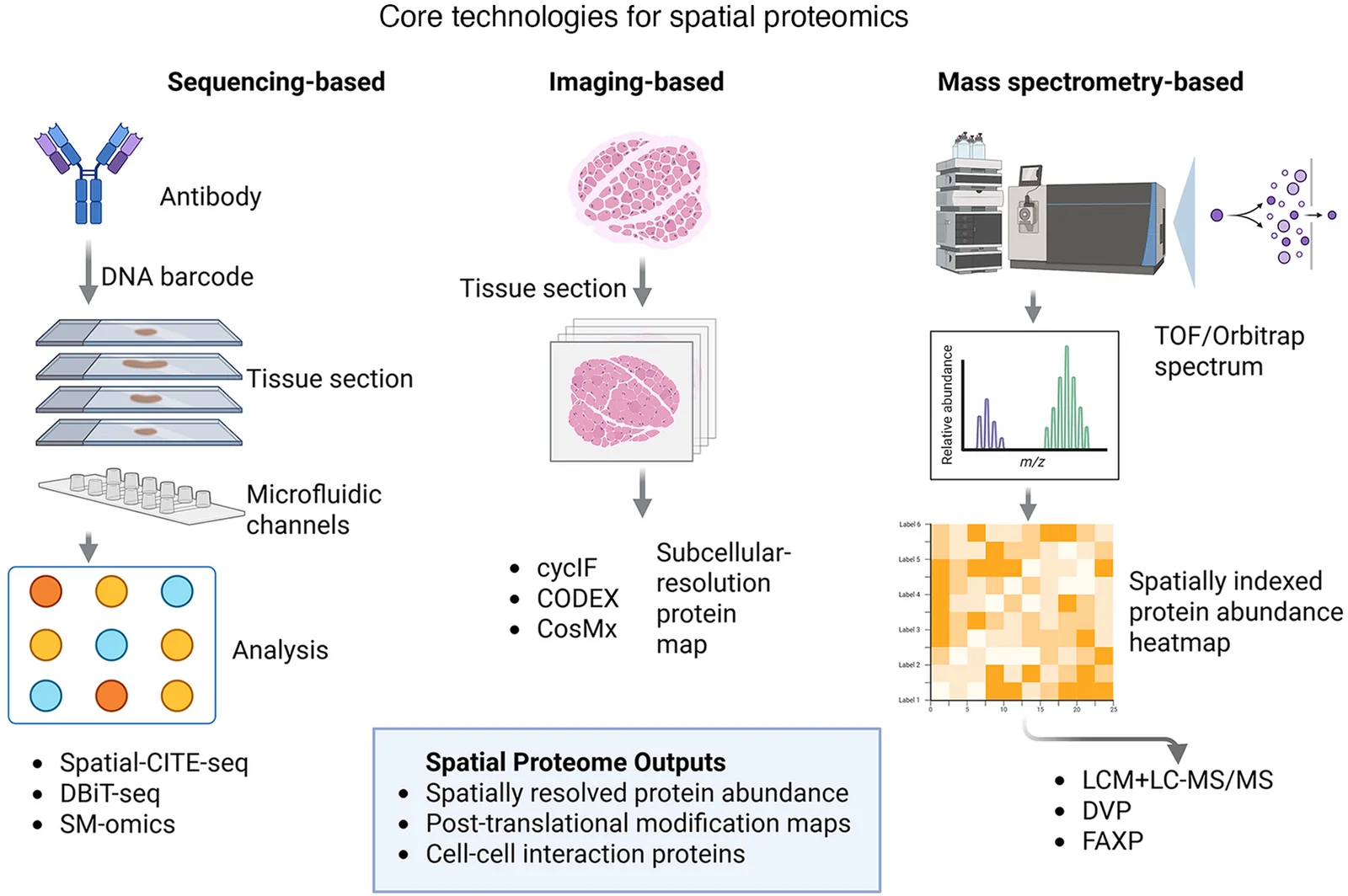
Core technologies for spatial proteomics. Schematic overview of the 3 major technological classes that enable spatially resolved proteome profiling in tissues. On the left, sequencing-based spatial proteomics uses oligonucleotide-barcoded antibodies — e.g., CITE-seq (67), cellular indexing of transcriptomes and epitopes by sequencing (spatial-CITE-seq) (12), spatially resolved CITE-seq (SM-omics) (68), and spatial multi-omics — to jointly capture RNA and protein signals, which are subsequently decoded through next-generation sequencing to provide spatially indexed protein maps. The middle panel shows imaging-based approaches — e.g., cyclic immunofluorescence (cycIF); co-detection by indexing (CODEX), CosMx (69), and CosMx spatial molecular imager; iterative bleaching extends multiplexing (IBEX) (20); and imaging mass cytometry (IMC) (70) — which rely on iterative staining, imaging, and signal cycling or multiplexed ion-based detection to achieve high-plex, subcellular-resolution protein localization. On the right, MS-based spatial proteomics integrates tissue microdissection or imaging MS — e.g., laser capture microdissection coupled with liquid chromatography–tandem MS (LCM-LC-MS/MS), deep visual proteomics (DVP) (73), and filter-aided expanded proteomics (FAXP) (74) — to spatially quantify thousands of proteins. TOF, time of flight. Collectively, these platforms enable reconstruction of spatial protein abundance, posttranslational modification patterns, and cell-cell interaction landscapes across tissue architectures.

Posttranscriptional regulation is crucial in cell fate determination and disease progression, underscoring the importance of proteomic profiling within intact tissue architecture. The rapid evolution of spatial proteomics has yielded a diverse technological landscape, encompassing sequencing-based, imaging-based, and MS-based strategies (Figure 3).

CITE-seq is a representative sequencing-based proteomics method that enables concurrent quantification of transcripts and dozens of surface proteins through oligonucleotide-barcoded Abs, offering an initial layer of multimodal integration (67). Spatial extensions, such as spatial-CITE-seq (12) and SM-omics (68), integrate RNA and protein detection through microfluidic barcoding platforms like DBiT-seq. These approaches highlight the potential of spatially resolved proteogenomics to capture dynamic cell-cell interactions and functional remodeling within intact tissues.

Complementary to sequencing-based approaches, imaging-based technologies offer direct visualization of protein localization at subcellular resolution. Commercial systems such as CosMx (69) enable simultaneous detection of dozens of protein markers through iterative staining and imaging cycles. Meanwhile, methods like IBEX (20) and imaging mass cytometry (IMC) (70), further expand multiplex capacity and enhance signal sensitivity. These technologies have enabled transformative insights in oncology. For example, highly multiplexed IMC revealed conserved spatial architectures of immune infiltration across breast cancer subtypes, defining reproducible tumor-immune microenvironment patterns associated with clinical outcome (70). Thus, spatial proteomics can serve as a framework for decoding functional tissue ecosystems rather than merely cataloging cell types. Moreover, multiplexed ion beam imaging (MIBI) identified distinct tumor-immune architectures in triple-negative breast cancer that were strongly associated with clinical outcome, highlighting the value of spatial protein biomarkers for risk stratification (71).

MS-based methods have markedly enhanced the depth of spatial proteomics analysis, enabling unbiased detection of thousands of proteins in a spatially resolved manner. LCM coupled with LC-MS/MS (LCM-MS) (72) has facilitated region-specific proteome profiling of glomeruli, tubules, and fibrotic environments. Meanwhile, more advanced strategies, such as Deep Visual Proteomics (DVP) (73) and filter-aided expansion proteomics (FAXP) (74), extend quantitative accuracy and FFPE compatibility. Beyond imaging-based platforms, integrative workflows such as DVP have bridged histopathology with unbiased MS, enabling cell-type-resolved proteome profiling directly from tissue sections (73). This approach demonstrates how morphology-guided proteomics can uncover disease-associated signaling programs within spatially defined niches, establishing a translational interface between digital pathology and systems proteomics.

Kühl et al. (75) developed a pathology-oriented multiplexing (PathoPlex) framework that integrates highly multiplexed subcellular-resolution imaging with computational analysis to extract biologically interpretable protein coexpression clusters across tissue architectures.

Despite these advances, direct mapping of protein-protein interactions (PPIs) within intact tissues remains an underdeveloped area of spatial multiomics (76). Current spatial proteomics platforms primarily quantify protein abundance and localization, whereas the spatial organization of signaling complexes and molecular interaction networks is largely inferred rather than directly measured. Recent work using multiplexed sequential proximity ligation assays has demonstrated the feasibility of spatially resolved subcellular PPI profiling, enabling reconstruction of signaling interactomes in both cultured cells and tissues (77). These emerging approaches may help bridge the gap between protein localization and functional interaction networks within native tissue microenvironments.

Collectively, spatial proteomics technologies have started to reveal region-specific protein composition, posttranslational modifications, and metabolic enzyme activity that cannot be inferred from transcriptomic data alone (Figure 3). Integration of spatial proteomics with transcriptomic and epigenomic maps will be essential for constructing multimodal tissue atlases that bridge regulatory potential with protein-level functional output. Ultimately, spatial proteomics provides a functional layer for decoding microenvironmental signaling, cellular heterogeneity, and disease-associated remodeling across organ systems.

## Spatial metabolomics and functional phenotyping

Spatial metabolomics has become a critical complement to transcriptomic and proteomic profiling that provides direct insights into cellular functional states within intact tissue environments. Unlike static molecular maps, metabolite distributions reflect real-time biochemical activity of cells and their dynamic responses to stress, hypoxia, and therapeutic intervention.

**Figure 4:**
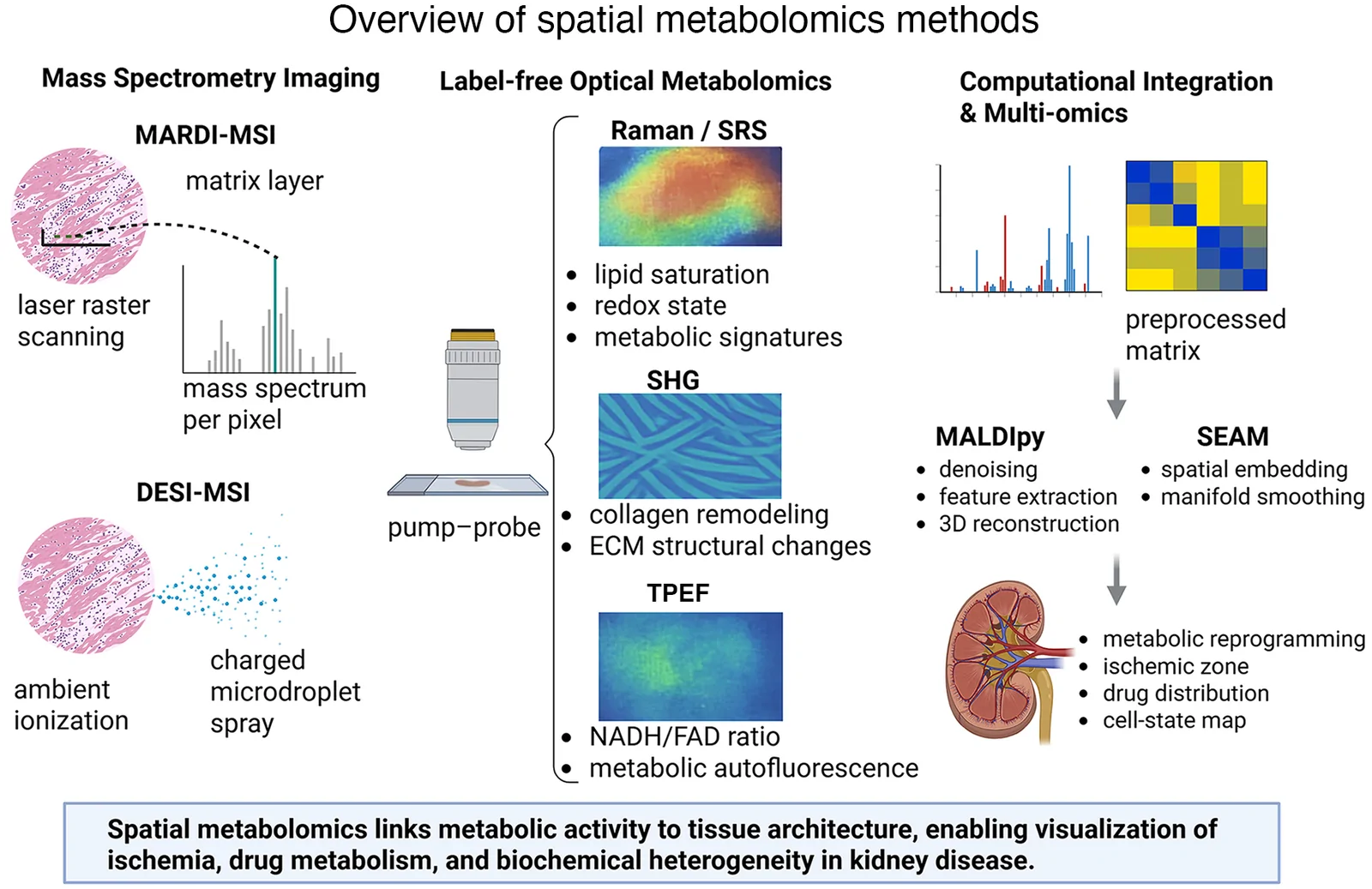
Overview of spatial metabolomics methods. On the left, MS imaging (MSI) approaches, including matrix-assisted laser desorption/ionization MSI (MALDI-MSI) and desorption electrospray ionization MSI (DESI-MSI), acquire pixel-resolved mass spectra to generate spatially resolved metabolite ion maps. In the middle, label-free optical metabolomics, such as Raman and stimulated Raman scattering (SRS), second harmonic generation (SHG) microscopy, and two-photon fluorescence (TPEF), captures lipid and redox signatures, ECM architecture, and autofluorescence-based metabolic features. On the right, computational pipelines integrate preprocessed intensity matrices with manifold-based spatial embedding and multivariate modeling to derive spatially resolved maps of metabolic reprogramming, ischemic niches, drug distribution, and cell-state organization.

Among available techniques, MSI, including DESI and MALDI, represents the cornerstone of spatial metabolomics (Figure 4). MSI acquires a full mass spectrum at each pixel, allowing ion intensities of metabolites or small molecules to be reconstructed into molecular images. In MALDI-MSI (21), a spatially confined laser desorption/ionization process enables micron-level localization of metabolites, during which specialized matrix deposition and tissue sectioning strategies preserve the original tissue structure. In contrast, DESI-MSI (22) directly ionizes analytes on tissue surfaces under ambient conditions without the need for matrix embedding.

These label-free technologies capture hundreds of lipids and small metabolites directly from tissue sections with micrometer-scale resolution, preserving both spatial and molecular contexts. In oncology, MALDI-MSI has uncovered spatially restricted metabolic programs within tumor ecosystems. For example, spatial lipidomic profiling of human glioblastoma demonstrated region-specific accumulation of phospholipid species and metabolic heterogeneity associated with hypoxic tumor niches (78). More broadly, MSI has enabled mapping of tumor-stroma metabolic interactions and oncometabolite gradients across multiple cancer types, establishing spatial metabolomics as a tool for resolving metabolic ecosystem architecture in situ. Spatial metabolomics has also contributed to efforts to generate systematic tissue atlases. For instance, comprehensive MALDI-MSI mapping of human organs has reconstructed spatial lipid distributions across multiple tissues, revealing conserved metabolic zonation patterns in liver and brain that are invisible in bulk metabolomics (79).

Beyond single-modality omics, multimodal integration is advancing toward the simultaneous capture of both protein and metabolite information. For example, scSpaMet (80) integrates single-cell proteomics with metabolite imaging, revealing functional coupling between metabolic states and protein expression signatures. This advance illustrates how spatial metabolomics can resolve functional immune microenvironments beyond transcript-level inference.

Recent clinical applications have demonstrated the translational potential of spatial metabolomics. In translational medicine, MSI has been widely used to visualize drug distribution and metabolism directly within tissues. Spatial mapping of chemotherapeutic agents and their metabolites has revealed heterogeneous intratumoral drug penetration and spatially restricted pharmacodynamic responses (81). These applications position spatial metabolomics as a bridge between molecular pathology and spatial pharmacology. Notably, recent advances in label-free optical imaging have demonstrated that multimodal Raman-based platforms that integrate stimulated Raman scattering, second harmonic generation, and two-photon fluorescence can simultaneously capture morphological, lipidomic, and metabolic biomarkers in diabetic kidneys at subcellular resolution (23) (Figure 4). This label-free optical biopsy approach bridges molecular and structural data, highlighting lipid saturation, redox state, and collagen remodeling as key spatially resolved indicators of diabetic nephropathy progression (23). Collectively, these advances demonstrate that spatial metabolomics provides a dynamic biochemical layer that complements transcriptomic and proteomic maps, enabling functional interpretation of tissue heterogeneity and drug responses across diverse physiological and pathological contexts.

## Computational overview of spatial multiomics analysis

With the rapid expansion of spatial multiomics technologies, platform-specific analytical pipelines have become increasingly standardized (Table 4). These pipelines now offer comprehensive end-to-end solutions, encompassing barcode decoding, spatial gene matrix reconstruction, and downstream domain identification. Barcoded microfluidic systems, such as DBiT-seq (9), rely on image-guided X/Y stripe registration, unique molecular identifier correction, and pixel-level matrix assembly. In contrast, bead-based platforms, like Slide-seqV2 (82), emphasize precise bead localization, barcode index decoding, and denoising or spatial deconvolution. The Visium workflow (Space Ranger) (83) further integrates histology-guided spot detection and cell-type deconvolution. Array-seq (11) extends this paradigm by enabling coordinate-defined decoding over large surfaces and 3D reconstructions from serial sections. Collectively, these pipelines establish the core computational foundations necessary for converting raw spatial signals into structured multiomic maps.

Beyond platform-specific workflows, a diverse array of general spatial analysis methods has emerged to support data integration, mapping, and domain inference across different omics layers (Table 5). Joint latent models, such as Seurat weighted nearest neighbor (WNN) (84), enable harmonized embedding of single-cell and spatial datasets. Meanwhile, mapping frameworks, like Tangram (85) and ENVI (86), align scRNA-seq profiles to spatial coordinates. Graph-based models, such as SpaGCN (87) and GraphST (88), leverage tissue morphology to enhance spatial resolution. In parallel, multiomic frameworks, like SpatialGlue (89), extend these capabilities to epigenomic and proteomic modalities.

For spatial metabolomics data analysis, computational frameworks, such as MALDIpy (90) and spatially enhanced analysis of metabolomics (SEAM) (91), were developed to improve data analysis and integration. These tools facilitate denoising, feature extraction, and 3D reconstruction of metabolic maps (90). These frameworks also enable co-registration with histological and proteomic data layers, providing a comprehensive and holistic view of tissue architecture and function. Complementary tools, including METASPACE (92) and SEAM (91), further expand the computational toolbox of spatial multiomics analysis (Table 5).

Despite the rapid development of computational frameworks, several major challenges remain (76). First, normalization across platforms remains difficult because different spatial multiomics technologies exhibit substantial variability in capture efficiency, spatial resolution, and signal-to-noise characteristics. Second, cross-modality integration is complicated by the distinct data structures and sparsity patterns of transcriptomic, epigenomic, proteomic, and metabolomic datasets, often requiring modality-specific assumptions that may introduce bias. Third, the lack of standardized benchmarking datasets and evaluation metrics hinders objective comparisons among computational methods. Future efforts should focus on developing robust normalization strategies, scalable multimodal integration frameworks, and community-wide benchmarking standards to improve reproducibility and facilitate clinical translation of spatial multiomics analyses (2).

## AI-driven virtual spatial profiling

Despite the promising future of spatial omics, multiple obstacles remain for its translation to large-scale clinical cohorts, including that it remains hindered by its prohibitive cost, intricate technical complexity, and stringent requirements for sample integrity. To circumvent these bottlenecks, virtual spatial profiling powered by multimodal AI foundation models has rapidly come to the forefront. Researchers have sought to bypass cost-intensive procedures by computational inference from readily accessible molecular data or by generating virtual omics profiles directly from widely available, routine modalities.

Routinely produced at low cost, hematoxylin and eosin–stained (H&E-stained) histopathology images can be highly integrated into clinical workflows. These images are effective for studying tissue architecture and cellular morphology, but they lack the molecular granularity to resolve detailed molecular pathways. Therefore, leveraging deep learning to predict spatial gene expression directly from H&E-stained slices has emerged as a powerful, scalable alternative.

Zhang et al. developed Thor (93), a comprehensive platform for cell-level ST and histology image analysis. Thor infers single-cell spatial transcriptomic profiles from coarse spot-level data through an anti-shrinking Markov diffusion method. This platform integrates histological sections and enables in-depth, multi-module downstream analysis that demonstrates accuracy and robustness across diverse tissue types.

A multitude of computational models have recently sprung up to that predict spatial gene expression from H&E-stained sections. STimage (94) applies ensemble learning to uncertainty quantification and gene expression distributions to enhance robustness, and it adopts the local interpretable model-agnostic explanations (LIME) algorithm, Cellpose-3 segmentation, and an adapted HoVer-Net architecture to attain single-cell interpretability. Path2Space (95), optimized through massive training on breast cancer ST data, delivers robust spatial expression predictions for thousands of genes and eclipses 21 other methods. Constructed on 41-plex spatial proteomic profiles from 457 patients with non–small cell lung cancer, CANVAS (96) is capable of predicting spatial habitat structures anchored in cellular neighborhoods, thereby facilitating clinical evaluations across data from more than 5,000 patients encompassing nine distinct cancer types. All these models demonstrate remarkable robustness and reliability in prognostic risk stratification and therapeutic response prediction. GigaTIME (97) can generate virtual multiplex IF (mIF) images from standard H&E-stained sections through large-scale training with paired H&E and mIF data. In Valanarasu et al. (97), GigaTIME was conducted on 14,256 patients and successfully uncovered 1,234 significant associations that bridge protein activation, biomarkers, pathological stages, and survival. Furthermore, a comprehensive “GigaTIME signature” integrated the virtual activation profiles across 21 protein channels and markedly outperformed any individual virtual channel in predicting patient survival trajectories and executing risk stratification.

Furthermore, generative AI has displayed irreplaceable advantages in the data integration and fidelity evaluation of in vitro model systems such as organoid models. To address the substantial batch effects driven by developmental and technical discrepancies between in vitro organoids and in vivo primary tissues, deep generative models based on conditional variational autoencoders (CVAEs) have proven highly effective in establishing non-linear latent space mapping and cross-system alignment (98). Generative adversarial networks (GANs) haves opened new additional avenues for the scalable and efficient integration of unpaired single-cell and spatial multiomics data, significantly enhancing the precision of cross-modal data fusion (99). Biomedical foundation models (BMFMs) are pretrained on large-scale single-cell transcriptomic data and capture universal cell-state representations via generative reconstruction tasks, thereby enabling the high-throughput quantitative benchmarking of the multidimensional physiological fidelity of in vitro culture systems for the first time (100).

## Conclusions

The functional complexity of an organ is dictated by its cellular and structural heterogeneity, which also provides crucial clues for understanding pathophysiology. Considering the major limitation of single-cell omics is the lack of positional mapping, spatial technologies represent a milestone in omics research by preserving the spatial context.

However, spatial multiomics remains in its infancy. Most commercialized platforms still lack true single-cell resolution, while high-resolution methods often sacrifice whole-genome or whole-transcriptome coverage. Moreover, many current studies depend on computational integration of multimodal data acquired from serial tissue sections, introducing potential misalignment and bias. Despite remarkable advances in spatial resolution and molecular coverage, accurate cell segmentation remains a major challenge across many ST platforms (2, 101). In imaging-based approaches, densely packed tissues, irregular cell morphologies, and overlapping cellular boundaries can compromise transcript assignment and introduce errors in cell-type identification. Similarly, sequencing-based methods often require computational deconvolution to resolve mixed-cell signals within capture regions. As spatial datasets continue to increase in complexity and resolution, robust segmentation algorithms and standardized benchmarking frameworks will be critical for improving data accuracy and biological interpretation (102).

Despite these limitations, pioneering spatial multiomics investigations have already yielded multifaceted insights into the intricate molecular networks governing organ organization and dysfunction. A fully integrated, unbiased approach that captures multiple layers simultaneously at high resolution holds great promise for understanding organ and tissue architecture and function. We envision that spatial genomics reveals driver mutations; spatial epigenomics delineates dynamic chromatin states that define cell identity and plasticity; spatial proteomics uncovers posttranscriptional regulation, posttranslational modifications, and shifts in metabolic machinery; and spatial metabolomics delivers direct functional readouts of cellular activity. By preserving spatial context across these modalities, spatial multiomics can illuminate emergent cellular phenotypes, niche architecture, and intercellular crosstalk within intact microenvironments.

Furthermore, future spatial multiomics technologies will move toward lower cost, higher throughput, and broader tissue coverage. Approaches will integrate additional modalities, and most importantly, transition from 2D to 3D. This shift will eliminate section artifacts and provide in-depth information of the tissue (103). Several attempts have been made in this direction. For instance, CODA reconstructed the cell-resolved 3D transition from pancreatic preneoplastic lesions to ductal adenocarcinoma using serially sectioned H&E-stained images. The workflow automatically categorized up to 10 distinct cell and tissue types without additional staining by leveraging deep learning semantic segmentation. This comprehensive matrix enabled precise, multiscale quantification of compositional and density shifts throughout neoplastic progression (104). Another 3D-imaging approach, light sheet fluorescence microscopy (LSFM), has been utilized to map neurovascular connectivity of nephrons (105).

Beyond technological innovation, each spatial multiomics modality provides unique opportunities to address distinct biological and translational questions. Spatial genomics enables the localization of disease-driving genetic alterations, spatial epigenomics reveals regulatory programs underlying cell-state transitions, spatial proteomics identifies functional biomarkers and therapeutic targets, and spatial metabolomics captures dynamic metabolic adaptations associated with disease progression and treatment response. Together, these complementary layers facilitate biomarker discovery, patient stratification, and precision medicine applications.

In summary, the rapid maturation of spatial multiomics technologies promises to yield increasingly comprehensive and refined atlases in the coming years. By transcending the limitations of transcriptomics-alone approaches, these integrated platforms will capture coordinated dynamics across genomics, epigenomics, transcriptomics, proteomics, and metabolomics layers within intact-tissue contexts. This deeper, multimodal resolution is poised to uncover novel biomarkers and therapeutic targets, illuminate patient-specific molecular trajectories and metabolic reprogramming, and pave the way for true precision medicine. Ultimately, spatial multiomics is poised to advance our understanding of physiology and pathology, shifting the field from static cell atlases toward functional models that could lay the foundation for future predictive and therapeutic applications.

## Author contributions

XM, ZC, EJH, JL, JH, and HL wrote the manuscript. XM and ZC prepared the figures and tables. HL supervised the project.

## Conflict of interest

The authors have declared that no conflict of interest exists.

## Funding support

- Shanghai Magnolia Talent Plan Pujiang Project (25PJA119).
- Internal research funding from Shanghai General Hospital.

## Supplementary Material

Supplemental data

## Figures and Tables

**Table 3:**
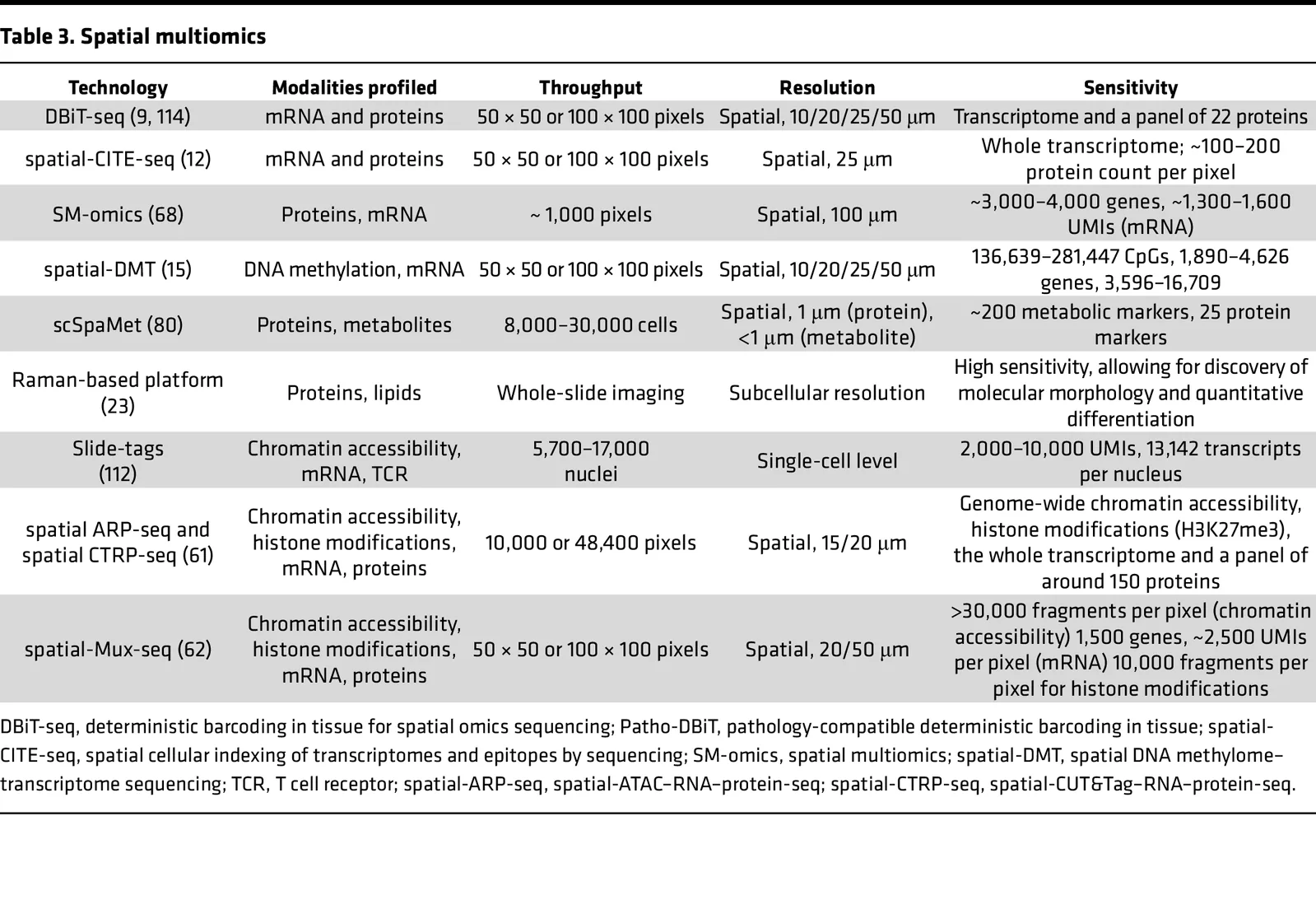
Spatial multiomics

**Table 1:**
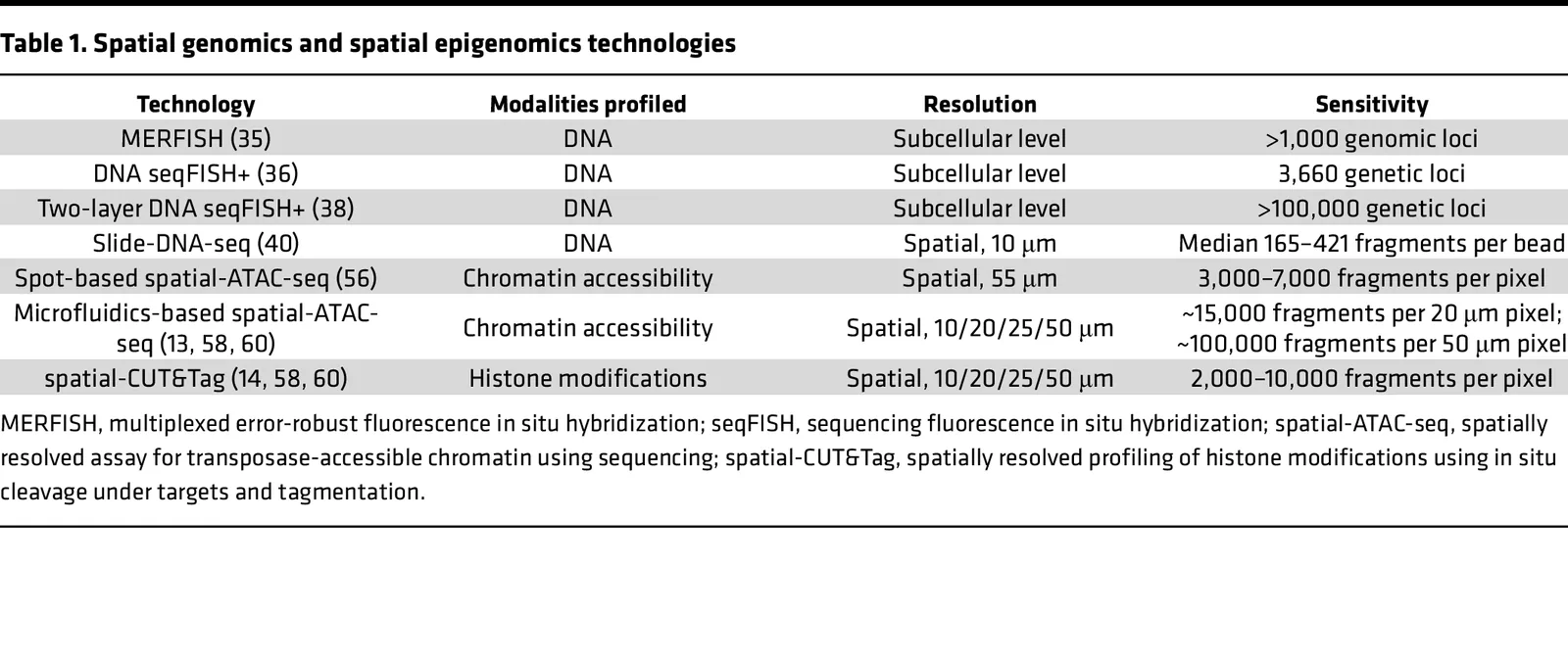
Spatial genomics and spatial epigenomics technologies

**Table 2:**
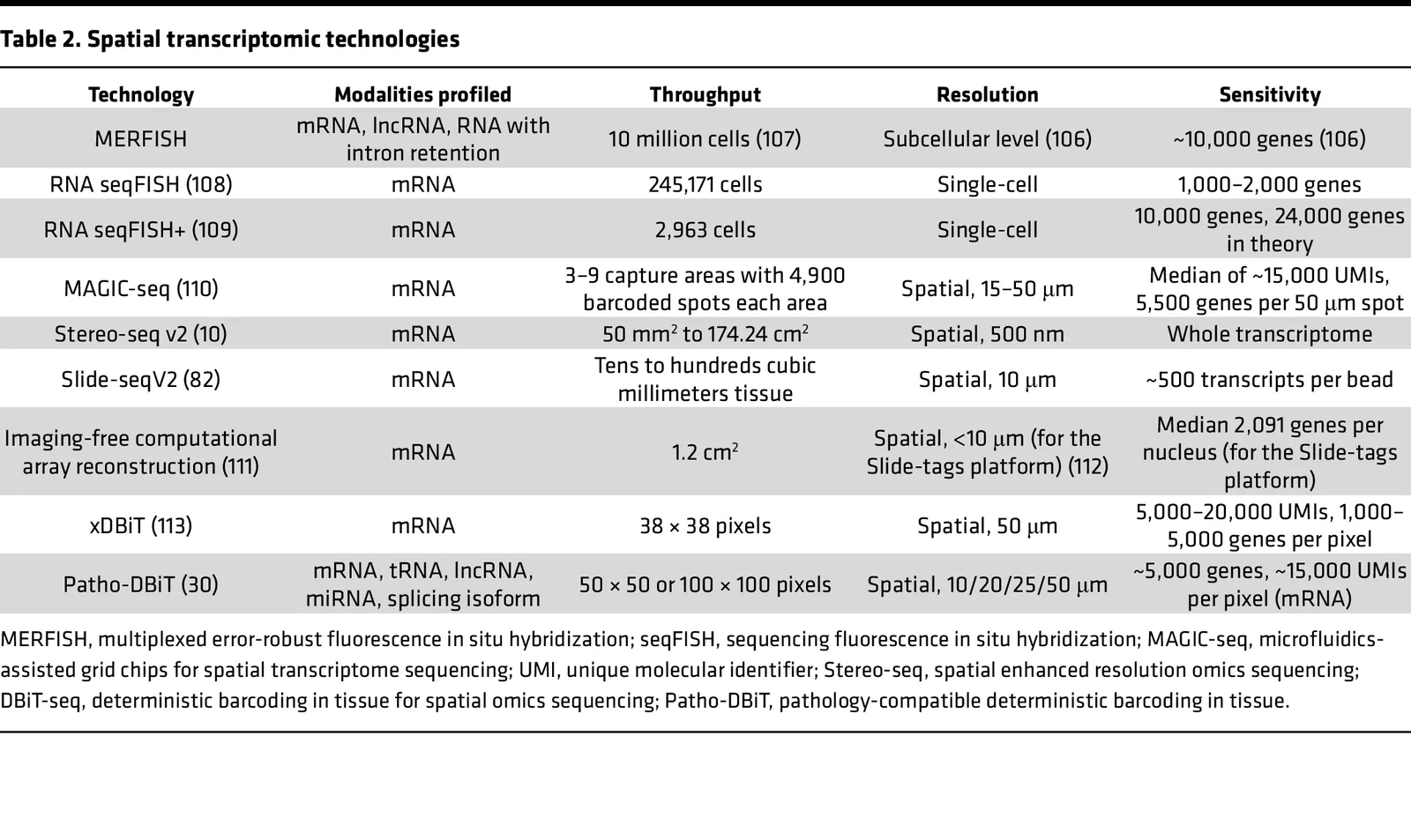
Spatial transcriptomic technologies

**Table 4:**
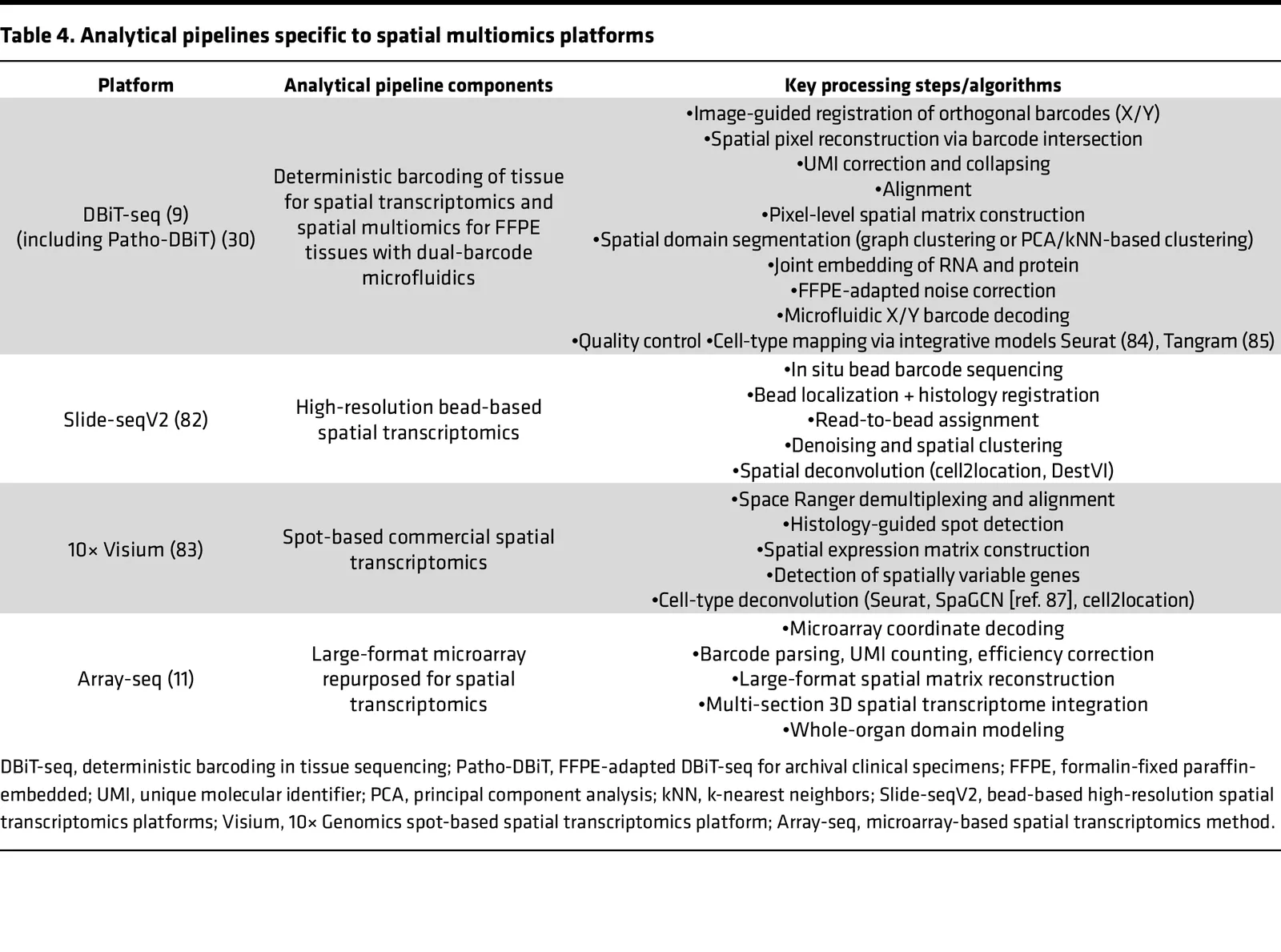
Analytical pipelines specific to spatial multiomics platforms

**Table 5:**
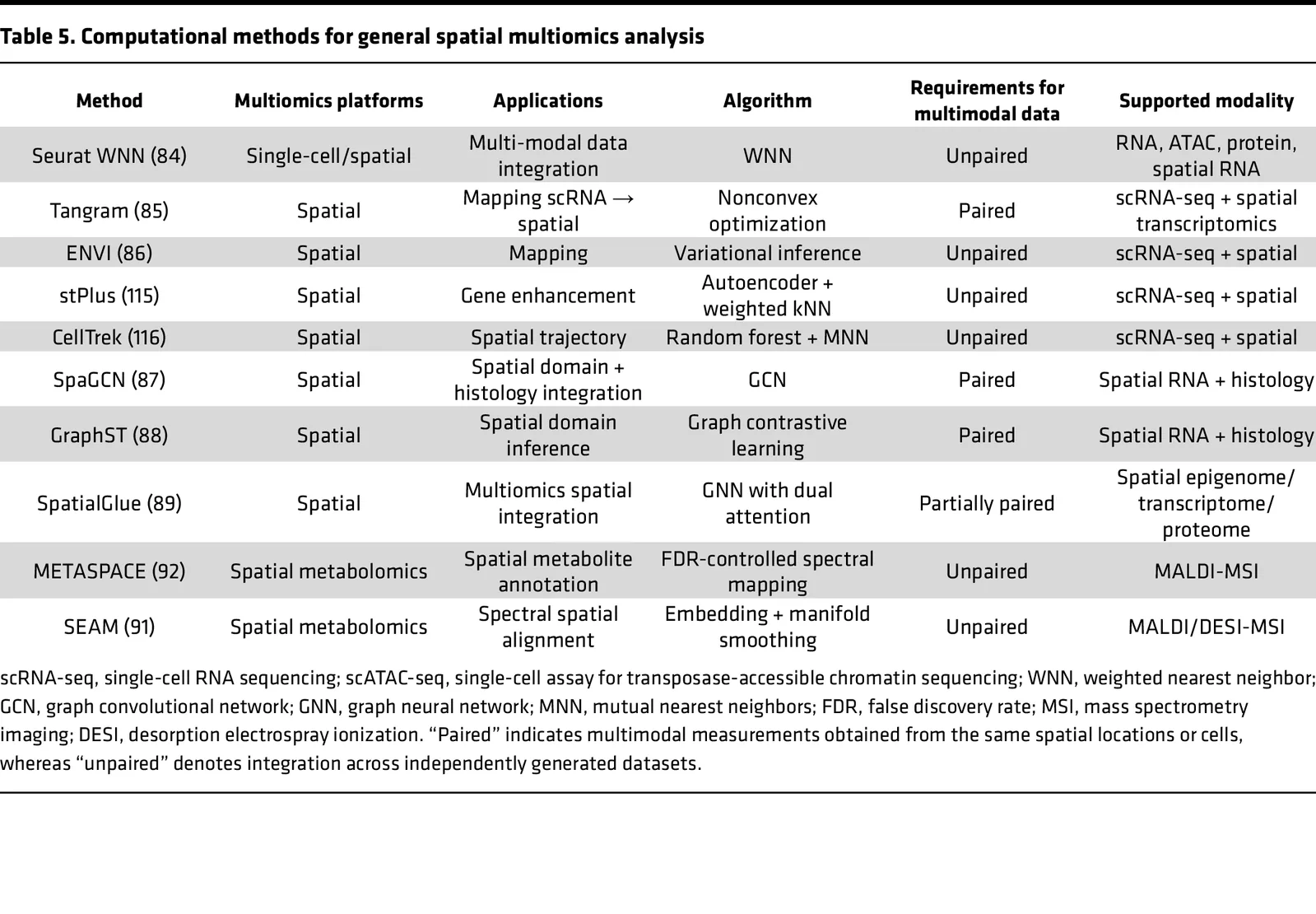
Computational methods for general spatial multiomics analysis

## Version 1 09/08/2026

Electronic publication

## References

[1] Baysoy A (2023). The technological landscape and applications of single-cell multi-omics. Nat Rev Mol Cell Biol.

[2] Moses L, Pachter L (2022). Museum of spatial transcriptomics. Nat Methods.

[3] Schwanhausser B (2011). Global quantification of mammalian gene expression control. Nature.

[4] Sharma K (2026). Spatial metabolomics and multiomics integration for breakthroughs in precision medicine for kidney disease. Nat Rev Nephrol.

[5] Mangiameli SM (2023). Photoselective sequencing: microscopically guided genomic measurements with subcellular resolution. Nat Methods.

[6] Haase C (2022). Image-seq: spatially resolved single-cell sequencing guided by in situ and in vivo imaging. Nat Methods.

[7] Oliveira MF (2025). High-definition spatial transcriptomic profiling of immune cell populations in colorectal cancer. Nat Genet.

[8] Chen A (2022). Spatiotemporal transcriptomic atlas of mouse organogenesis using DNA nanoball-patterned arrays. Cell.

[9] Liu Y (2020). High-spatial-resolution multi-omics sequencing via deterministic barcoding in tissue. Cell.

[10] Zhao Y (2025). Stereo-seq v2: spatial mapping of total RNA on FFPE sections with high resolution. Cell.

[11] Cipurko D (2025). Repurposing large-format microarrays for scalable spatial transcriptomics. Nat Methods.

[12] Liu Y (2023). High-plex protein and whole transcriptome co-mapping at cellular resolution with spatial CITE-seq. Nat Biotechnol.

[13] Deng Y (2022). Spatial profiling of chromatin accessibility in mouse and human tissues. Nature.

[14] Yanxiang Deng MB (2022). Spatial-CUT&Tag: Spatially resolved chromatin modification profiling at the cellular level. Science.

[15] Lee CN (2025). Spatial joint profiling of DNA methylome and transcriptome in tissues. Nature.

[16] Alon S (2021). Expansion sequencing: spatially precise in situ transcriptomics in intact biological systems. Science.

[17] Zeng H (2023). Integrative in situ mapping of single-cell transcriptional states and tissue histopathology in a mouse model of Alzheimer’s disease. Nat Neurosci.

[18] Nguyen HQ (2020). 3D mapping and accelerated super-resolution imaging of the human genome using in situ sequencing. Nat Methods.

[19] Li Z (2024). Presence of onco-fetal neighborhoods in hepatocellular carcinoma is associated with relapse and response to immunotherapy. Nat Cancer.

[20] Radtke AJ (2022). IBEX: an iterative immunolabeling and chemical bleaching method for high-content imaging of diverse tissues. Nat Protoc.

[21] Neumann EK (2020). Spatial metabolomics of the human kidney using MALDI trapped ion mobility imaging mass spectrometry. Anal Chem.

[22] Wang Z (2021). Spatial-resolved metabolomics reveals tissue-specific metabolic reprogramming in diabetic nephropathy by using mass spectrometry imaging. Acta Pharm Sin B.

[23] Fung AA (2025). Label-free multimodal optical biopsy reveals biomolecular and morphological features of diabetic kidney tissue in 2D and 3D. Nat Commun.

[24] Marco Salas S (2025). Optimizing xenium in situ data utility by quality assessment and best-practice analysis workflows. Nat Methods.

[25] Borm LE (2023). Scalable in situ single-cell profiling by electrophoretic capture of mRNA using EEL FISH. Nat Biotechnol.

[26] Wan Y (2026). Whole-embryo spatial transcriptomics at subcellular resolution from gastrulation to organogenesis. Science.

[29] Fu X (2022). Polony gels enable amplifiable DNA stamping and spatial transcriptomics of chronic pain. Cell.

[30] Bai Z (2024). Spatially exploring RNA biology in archival formalin-fixed paraffin-embedded tissues. Cell.

[31] Enninful A Integration of imaging-based and sequencing-based spatial omics mapping on the same tissue section via DBiTplus. Nat Methods.

[32] Gebert LFR, MacRae IJ (2019). Regulation of microRNA function in animals. Nat Rev Mol Cell Biol.

[33] Kim Y (2025). Seq-Scope: repurposing Illumina sequencing flow cells for high-resolution spatial transcriptomics. Nat Protoc.

[34] Schott M (2024). Open-ST: high-resolution spatial transcriptomics in 3D. Cell.

[35] Su JH (2020). Genome-scale imaging of the 3D organization and transcriptional activity of chromatin. Cell.

[36] Takei Y (2021). Integrated spatial genomics reveals global architecture of single nuclei. Nature.

[37] Lieberman-Aiden E (2009). Comprehensive mapping of long-range interactions reveals folding principles of the human genome. Science.

[38] Takei Y (2025). Spatial multi-omics reveals cell-type-specific nuclear compartments. Nature.

[39] Liu L (2024). Spatiotemporal omics for biology and medicine. Cell.

[40] Zhao T (2022). Spatial genomics enables multi-modal study of clonal heterogeneity in tissues. Nature.

[41] Kim J (2023). Barcoded multiple displacement amplification for high coverage sequencing in spatial genomics. Nat Commun.

[42] Payne AC (2021). In situ genome sequencing resolves DNA sequence and structure in intact biological samples. Science.

[43] Labade AS (2025). Expansion in situ genome sequencing links nuclear abnormalities to aberrant chromatin regulation. Science.

[44] Steinmetz LM, Davis RW (2004). Maximizing the potential of functional genomics. Nat Rev Genet.

[45] Dixit A (2016). Perturb-Seq: dissecting molecular circuits with scalable single-cell RNA profiling of pooled genetic screens. Cell.

[46] Dhainaut M (2022). Spatial CRISPR genomics identifies regulators of the tumor microenvironment. Cell.

[47] Saunders RA (2025). Perturb-multimodal: a platform for pooled genetic screens with imaging and sequencing in intact mammalian tissue. Cell.

[48] Breinig M (2026). Integrated in vivo combinatorial functional genomics and spatial transcriptomics of tumours to decode genotype-to-phenotype relationships. Nat Biomed Eng.

[49] Mollaoglu G (2024). Ovarian cancer-derived IL-4 promotes immunotherapy resistance. Cell.

[50] Baysoy A Large-scale, spatially resolved panoramic CRISPR screening in native tissue environments using Perturb-DBiT. Nat Biotechnol.

[51] Carter B, Zhao K (2021). The epigenetic basis of cellular heterogeneity. Nat Rev Genet.

[52] Wen L, Tang F (2022). Recent advances in single-cell sequencing technologies. Precis Clin Med.

[53] Buenrostro JD (2015). ATAC-seq: a method for assaying chromatin accessibility genome-wide. Curr Protoc Mol Biol.

[55] Carraro C (2023). Chromatin accessibility profiling of targeted cell populations with laser capture microdissection coupled to ATAC-seq. Cell Rep Methods.

[56] Llorens-Bobadilla E (2023). Solid-phase capture and profiling of open chromatin by spatial ATAC. Nat Biotechnol.

[58] Li H (2025). Spatially resolved genome-wide joint profiling of epigenome and transcriptome with spatial-ATAC-RNA-seq and spatial-CUT&Tag-RNA-seq. Nat Protoc.

[59] Jiang F (2023). Simultaneous profiling of spatial gene expression and chromatin accessibility during mouse brain development. Nat Methods.

[60] Zhang D (2023). Spatial epigenome-transcriptome co-profiling of mammalian tissues. Nature.

[61] Zhang D (2025). Spatial dynamics of brain development and neuroinflammation. Nature.

[62] Guo P (2025). Multiplexed spatial mapping of chromatin features, transcriptome and proteins in tissues. Nat Methods.

[63] Guo P (2025). Spatial profiling of chromatin accessibility in formalin-fixed paraffin-embedded tissues. Nat Commun.

[64] Li H (2026). Spatially decoding genotype-associated epigenetic landscapes in human lymphoma FFPE tissues via epi-Patho-DBiT. Nat Commun.

[65] Chen F (2024). Visualizing epigenetic modifications and their spatial proximities in single cells using three DNA-encoded amplifying FISH imaging strategies: BEA-FISH, PPDA-FISH and Cell-TALKING. Nat Protoc.

[66] Lu T (2022). Spatially resolved epigenomic profiling of single cells in complex tissues. Cell.

[67] Stoeckius M (2017). Simultaneous epitope and transcriptome measurement in single cells. Nat Methods.

[68] Vickovic S (2022). SM-Omics is an automated platform for high-throughput spatial multi-omics. Nat Commun.

[69] He S (2022). High-plex imaging of RNA and proteins at subcellular resolution in fixed tissue by spatial molecular imaging. Nat Biotechnol.

[70] Jackson HW (2020). The single-cell pathology landscape of breast cancer. Nature.

[71] Keren L (2018). A structured tumor-immune microenvironment in triple negative breast cancer revealed by multiplexed ion beam imaging. Cell.

[72] Zemaitis KJ (2025). Spatial top-down proteomics for the functional characterization of human kidney. Clin Proteomics.

[73] Mund A (2022). Deep visual proteomics defines single-cell identity and heterogeneity. Nat Biotechnol.

[74] Dong Z (2024). Spatial proteomics of single cells and organelles on tissue slides using filter-aided expansion proteomics. Nat Commun.

[75] Kuehl M (2025). Pathology-oriented multiplexing enables integrative disease mapping. Nature.

[76] Burgess DJ (2019). Spatial transcriptomics coming of age. Nat Rev Genet.

[77] Cai S (2025). Spatially resolved subcellular protein-protein interactomics in drug-perturbed lung-cancer cultures and tissues. Nat Biomed Eng.

[78] Randall EC (2020). Localized metabolomic gradients in patient-derived xenograft models of glioblastoma. Cancer Res.

[79] Schwamborn K, Caprioli RM (2010). Molecular imaging by mass spectrometry--looking beyond classical histology. Nat Rev Cancer.

[80] Hu T (2023). Single-cell spatial metabolomics with cell-type specific protein profiling for tissue systems biology. Nat Commun.

[81] Nilsson A (2015). Mass spectrometry imaging in drug development. Anal Chem.

[82] Stickels RR (2021). Highly sensitive spatial transcriptomics at near-cellular resolution with Slide-seqV2. Nat Biotechnol.

[83] Du MRM (2025). Benchmarking spatial transcriptomics technologies with the multi-sample SpatialBenchVisium dataset. Genome Biol.

[84] Hao Y (2021). Integrated analysis of multimodal single-cell data. Cell.

[85] Biancalani T (2021). Deep learning and alignment of spatially resolved single-cell transcriptomes with Tangram. Nat Methods.

[86] Haviv D (2024). The covariance environment defines cellular niches for spatial inference. Nat Biotechnol.

[87] Hu J (2021). SpaGCN: Integrating gene expression, spatial location and histology to identify spatial domains and spatially variable genes by graph convolutional network. Nat Methods.

[88] Long Y (2023). Spatially informed clustering, integration, and deconvolution of spatial transcriptomics with GraphST. Nat Commun.

[89] Long Y (2024). Deciphering spatial domains from spatial multi-omics with SpatialGlue. Nat Methods.

[90] Li H (2024). Transcriptomic, epigenomic, and spatial metabolomic cell profiling redefines regional human kidney anatomy. Cell Metab.

[91] Yuan Z (2021). SEAM is a spatial single nuclear metabolomics method for dissecting tissue microenvironment. Nat Methods.

[92] Wadie B (2024). METASPACE-ML: context-specific metabolite annotation for imaging mass spectrometry using machine learning. Nat Commun.

[93] Zhang P (2025). Thor: a platform for cell-level investigation of spatial transcriptomics and histology. Nat Commun.

[94] Tan X (2026). Robust and interpretable prediction of gene markers and cell types from spatial transcriptomics data. Nat Commun.

[95] Shulman ED (2026). AI-predicted spatial transcriptomics unlocks breast cancer biomarkers from pathology. Cell.

[96] Li Y (2026). Cellular architecture and neighborhood-informed virtual spatial tumor profiling from histopathology. Cell.

[97] Valanarasu JMJ (2026). Multimodal AI generates virtual population for tumor microenvironment modeling. Cell.

[99] Valentina Giansanti FG (2024). Scalable integration of multiomic single-cell data using generative adversarial networks. Bioinformatics.

[100] Fujii S (2026). Quantifying the fidelity of in vitro human cell culture systems using a biomedical foundation model. Proc Natl Acad Sci U S A.

[101] Marx V (2021). Method of the year: spatially resolved transcriptomics. Nat Methods.

[102] Petukhov V (2022). Cell segmentation in imaging-based spatial transcriptomics. Nat Biotechnol.

[103] Li KYC (2024). Cryo-X-ray phase contrast imaging enables combined 3D structural quantification and nucleic acid analysis of myocardial biopsies. Adv Sci (Weinh).

[104] Kiemen AL (2022). CODA: quantitative 3D reconstruction of large tissues at cellular resolution. Nat Methods.

[105] McLaughlin L (2025). Three dimensional multiscalar neurovascular nephron connectivity map of the human kidney across the lifespan. Nat Commun.

[106] Xia C (2019). Spatial transcriptome profiling by MERFISH reveals subcellular RNA compartmentalization and cell cycle-dependent gene expression. Proc Natl Acad Sci U S A.

[107] Zhang M (2023). Molecularly defined and spatially resolved cell atlas of the whole mouse brain. Nature.

[108] Polonsky M (2024). Spatial transcriptomics defines injury specific microenvironments and cellular interactions in kidney regeneration and disease. Nat Commun.

[109] Eng CHL (2019). Transcriptome-scale super-resolved imaging in tissues by RNA seqFISH. Nature.

[110] Zhu J (2024). Custom microfluidic chip design enables cost-effective three-dimensional spatiotemporal transcriptomics with a wide field of view. Nat Genet.

[111] Chenlei H (2026). Scalable spatial transcriptomics through computational array reconstruction. Nat Biotechnol.

[112] Russell AJC (2024). Slide-tags enables single-nucleus barcoding for multimodal spatial genomics. Nature.

[113] Wirth J (2023). Spatial transcriptomics using multiplexed deterministic barcoding in tissue. Nat Commun.

[114] Ge Q (2025). Enhancing RNA capture efficiency in spatial transcriptomics: a review of innovative technologies and strategies. Int J Mol Sci.

[115] Shengquan C (2021). stPlus: a reference-based method for the accurate enhancement of spatial transcriptomics. Bioinformatics.

[116] Wei R (2022). Spatial charting of single-cell transcriptomes in tissues. Nat Biotechnol.

[117] Liu J (2023). Concordance of MERFISH spatial transcriptomics with bulk and single-cell RNA sequencing. Life Sci Alliance.

[118] Wang H (2025). Systematic benchmarking of imaging spatial transcriptomics platforms in FFPE tissues. Nat Commun.

[119] Lake BB (2023). An atlas of healthy and injured cell states and niches in the human kidney. Nature.

