# Supplementary material for: Spatial multiomics in biomedical research: advances beyond transcriptomics
Source: JCI Insight. 2026 Sep 8;11(17):e206951. doi: 10.1172/jci.insight.206951 (PMC13564081; doi:10.1172/jci.insight.206951)
Supplement: Supplementary file 1 [file jciinsight-11-206951-s097.pdf]

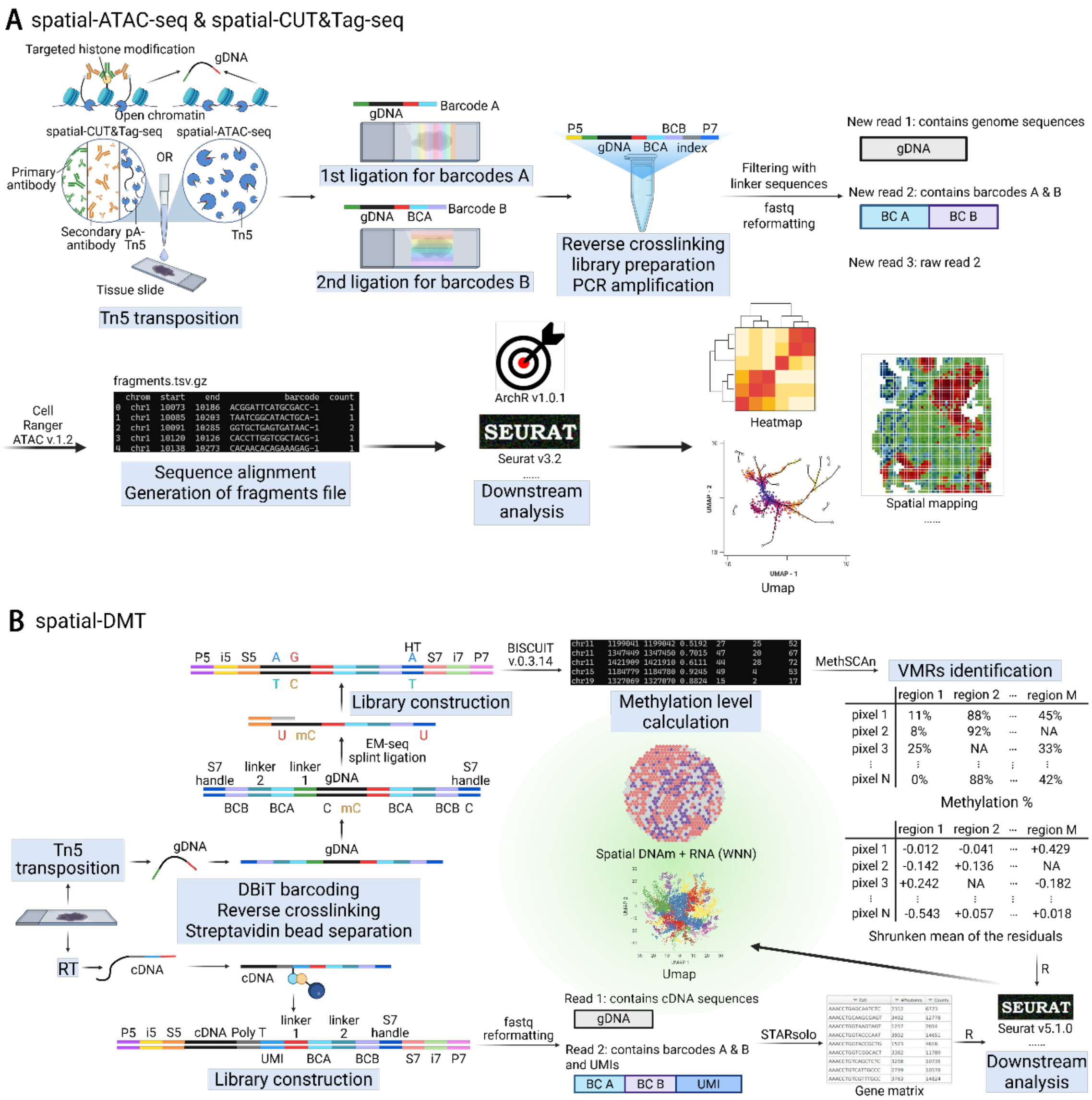

Supplementary Figure 1. The principles and data processing pipelines of spatial-ATAC-seq, spatial-CUT&Tag and spatial-DMT. BCA: Barcode A; BCB: Barcode B.

Supplementary Table 1. Cost, Applicability and Applications of spatial omics technologies

Part I: Imaging-based spatial omics technologies

Technology	Cost	Applicable sample types	Existing applications
**MERFISH**	Independent of gene/loci number; cost per unit decreases as multiplexity increases(35)<br>~$0.01 per cell at 10,000-gene scale (106)	Fresh frozen tissue (115); FFPE tissue (118)	1. Mapping the specific distribution of active promoters (66) and Whole-transcriptome-scale expression profiling (107) in the mouse brain<br>2. Identification of cell types and spatial structure in healthy murine kidney (115)<br>3. Spatially resolved cell typing of 17 tumor and 16 normal tissue types (118)
**seqFISH** (108)	Medium-High	Fresh frozen tissue	Defining injury specific microenvironments and cell-cell interactions in kidney regeneration and disease
**seqFISH+**	Medium-High	Fresh frozen tissue (109)	1. Whole genome imaging of mouse brain (38)<br>2. ST profiling of mouse brain (109)
**Raman-based platform** (24)	High	Fresh frozen tissue	Lipidome and other Metabolome in both normal and DN kidney tissues

Part II: Solid-phase array (bead/chip) spatial omics technologies

Technology	Cost	Applicable sample types	Existing applications
**Stereo-seq V2 (10)**	Medium-High	Fresh frozen tissue; FFPE tissue	1. Simultaneous host-pathogen transcriptome profiling in human tuberculous lung tissue<br>2. Spatial atlas of lncRNA in mouse brain.<br>3. Mapping tumor subtypes and alternative splicing in clinical breast cancer FFPE specimens archived for 9 years
**Slide-seq**	Medium	Fresh frozen tissue (40,82)	1. ST in mouse normal brain (82)<br>2. Tumor clones and genomic mutations in mouse model of liver matastasis and human metastatic colon cancer (40)<br>3. Spatially defining injury states and fibrotic niches in kidneys (119)
**Slide-tags** (112)	Medium	Fresh frozen tissue	1.ST in embryonic mouse brain and human cerebral cortex<br>2. Immune microenvironment and BCR–ligand interactions in the human tonsil<br>3. TME of human metastatic melanoma.
**SM-omics** (68)	High	Fresh frozen tissue	ST and proteomics in mouse brain, spleen and colorectal cancer model
**Spot-based spatial-ATAC-seq** (56)	High	Fresh frozen tissue	1. Mouse organogenesis<br>2. spatiotemporal atlas of epigenetic reprogramming in the mouse brain<br>3. TME epigenetic signatures in HER2+ human breast cancer

Part III: Solid-phase array (bead/chip) spatial omics technologies

Technology	Cost	Applicable sample types	Existing applications
**MAGIC-seq** (110)	Low; $0.11 per mm^2 for the chip fabrication	Fresh frozen tissue	High-throughput spatial transcriptomics of distinct mouse tissues

**DBiT-seq** (9,114)	Low-Medium; $1000 per tissue section	Fresh frozen tissue	ST and proteomics in mouse embryo sections
**xDBiT** (113)	Low-Medium; 125€ per tissue section	Fresh frozen tissue; FFPE tissue	ST in mouse cerebellum, liver, kidney, spleen and heart
**Patho-DBiT** (30)	Medium	FFPE tissue	Spatial whole RNA landscape, spatial SNV, and spatial distribution of splicing isoforms in human lymphoma FFPE samples
**Spatial-CITE-seq (12)**	High	Fresh frozen tissue	Spatial profiling of whole transcriptome and proteome in mouse tissues, human tonsil and skin.
**Microfluidics-based spatial-ATAC-seq** (13,58,60)	Medium	Fresh frozen tissue	1. Identifying of tissue domains and regulatory elements in mouse embryos, mouse brains, human hippocampi, and human tonsil (13,58,60)<br>2. Cell identification in mouse embryos and developmental trajectory tracing in the mouse brain (14)
**Spatial-CUT&Tag** (14,58,60)	Medium	Fresh frozen tissue	
**spatial-DMT (15)**	High	Fresh frozen tissue; FFPE tissue	1. Lineage tracing of mouse embryos<br>2. DNA methylation landscape of the mouse brain
**spatial ARP-seq & spatial CTRP-seq** (61)	High	Fresh frozen tissue	1. Spatiotemporal atlas of the epigenome, transcriptome, and proteome in the mammalian cerebral cortex<br>2. Developmental patterns of the corpus callosum<br>3. Dynamic pathophysiological changes in the focal demyelination injury model
**spatial-Mux-seq** (62)	High	Fresh frozen tissue	Multi-omics analysis of mouse embryo, brain, and EAE models

Part IV: Imaging-free computational array reconstruction

Technology	Cost	Applicable sample types	Existing applications
**Imaging-free computational array reconstruction** (106,111)	Low	Fresh frozen tissue	Identifying spatial cell clustering on a 1.2 cm^2 P1 mouse cranial section

Part V: Spatial metabolomics

Technology	Cost	Applicable sample types	Existing applications
**scSpaMet** (80)	Very high	Fresh frozen tissue; FFPE tissue	Metabolic heterogeneity in human lung cancer, tonsil and endometrium

Abbreviations: MERFISH, multiplexed error-robust fluorescence in situ hybridization; FFPE, formalin-fixed paraffin-embedded; seqFISH (+), sequencing fluorescence in situ hybridization (+); ST, spatial transcriptomics; FISSEQ, fluorescence in situ sequencing; MAGIC-seq, microfluidics-assisted grid chips for spatial transcriptome sequencing; Stereo-seq, spatial enhanced resolution omics sequencing; TCR, T cell receptor; BCR. B cell receptor; TME, tumor microenvironment; DBiT-seq, deterministic barcoding in tissue for spatial omics sequencing; Patho-DBiT, pathology-compatible deterministic barcoding in tissue; SNV, single nucleotide variant; spatial-CITE-seq, spatial cellular indexing of transcriptomes and epitopes by sequencing; SM-omics, spatial multiomics; spatial-ATAC-seq, spatially resolved assay for transposase-accessible chromatin using sequencing; spatial-CUT&Tag, spatially resolved profiling of histone modifications using in situ cleavage under targets and tagmentation; spatial-DMT, spatial DNA methylome–

transcriptome sequencing; spatial-ARP-seq, spatial-ATAC–RNA–protein-seq; spatial-CTRP-seq, spatial-CUT&Tag–RNA–protein-seq; EAE, experimental autoimmune encephalomyelitis; DN, diabetic nepfropathy.

Supplementary Table 2. Comparison of conventional transcriptomics and representative commercial spatial transcriptomics platforms

Feature	Bulk RNA-seq	scRNA-seq	Visium HD	Xenium
**Spatial information**	No	No	Yes (2D tissue mapping)	Yes (2D tissue mapping)
**Resolution**	Tissue	Single cell	~2 μm high-density grid (computational cell-typing required)	Subcellular (~0.2 μm optical resolution)
**Transcript coverage**	Whole transcriptome	Whole transcriptome	Whole transcriptome	Targeted panel
**Detection strategy**	Sequencing	Sequencing	Spatial barcoded sequencing	Imaging-based ISS
**Cell morphology**	NO (tissue homogenate)	NO (dissociated cells)	Histology-assisted	Direct imaging
**Cell segmentation**	Not required	Dissociation	Computational inference	Required
**FFPE compatibility**	Yes (with kit)	Limited (low RNA integrity)	Yes (probe-based)	Yes (optimized)
**Throughput (per run)**	High (multiple samples)	High (thousands of cells)	High (millions of spots, 2 areas/slide)	Moderate (area-limited, ~6.5×6.5 mm max)
**Major strengths**	Comprehensive profiling	Cell-type resolution	Genome-wide spatial profiling	High-resolution localization
**Major limitations**	No spatial context	Loss of spatial context	Cost; limited by segmentation/deconvolution methods	Cost; non-whole-genome; imaging time and segmentation challenges
**Representative applications**	Differential expression	Cell atlas	Tissue architecture	Cell-cell interaction
